# Participating in a parenting intervention in prison, perceptions from incarcerated fathers and mothers—A convergent mixed-methods study

**DOI:** 10.1371/journal.pone.0282326

**Published:** 2023-03-01

**Authors:** Åsa Norman, Pia Enebrink

**Affiliations:** Department of Clinical Neurosciences, Karolinska Institutet, Stockholm, Sweden; West University of Timisoara: Universitatea de Vest din Timisoara, ROMANIA

## Abstract

**Background:**

Children of incarcerated parents run a high risk of ill-health and future delinquency, whereas positive parenting can support children’s healthy development. The For Our Children’s Sake (FOCS) parenting intervention for parents in prison was evaluated as a controlled trial during 2019–2021 within The Swedish Prison and Probation Service (SPPS). This study reports on the process evaluation and aimed to describe how parents perceived their participation and aspects that influenced implementation of the FOCS intervention.

**Methods:**

This convergent mixed-methods study (QUAL + quan) included qualitative interview data after participation in the FOCS intervention group (12 parents), and quantitative questionnaire data from intervention and control groups (46 parents). Qualitative data were analysed using inductive qualitative content analysis and quantitative data using descriptive and non-parametric statistics.

**Results:**

An integrated synthesis of the qualitative and quantitative results showed three joint concepts that provided an extended understanding of *the importance of a child and parent focused intervention available to parents in prison*, where FOCS was perceived as the only place where inmates could openly reflect, and express sensitive feelings and thoughts related to the children and being a parent. Also, that the SPPS as an organisation entails *partly unsupportive organisational norms with irregular individual staff engagement*, which made FOCS invisible in prisons, and *the importance of engagement and motivation from all participants and group leaders in the group* was essential for a successful FOCS group.

**Conclusion:**

This study showed that availability of a child and parent focused intervention in prison is perceived as very important, and at the same time dependent on a trustful relationship in the group to be rewarding to the participants, where organisational norms within the SPSS need amendments for successful implementation of FOCS. These findings can guide further implementation of similar interventions in prison.

## Introduction

Positive parenting is essential to a healthy development process for all children [1, 2], and parent training has been found successful in preventing antisocial behaviour and delinquency [3]. Findings from both the international arena [4–6] as well as the Swedish context [6–8] specifically, have shown that children with a parent incarcerated in prison have an elevated risk for poor health in a number of areas, including poor well-being and behaviour problems such as antisocial behaviour, as well as future delinquency [6, 8, 9]. The development of delinquency in this child group has been suggested to be explained by intergenerational transmission of delinquent behaviour from the parent to the child [8, 9], and the criminally active parents may have difficulties in engaging in positive parenting strategies [10, 11]. Internationally developed interventions to support positive parenting for parents in prison have indicated positive influences on outcomes related to parenting [12–14], and improved child behaviours [13]. In Sweden, the parenting intervention For Our Children’s Sake (FOCS), which aims to support positive parenting for children’s healthy development, is delivered with fathers and mothers incarcerated in Swedish prisons. Effects of the FOCS intervention on parenting was evaluated as a controlled trial in 15 prisons (8 intervention, 7 control) during 2019–2021 [15]. Effectiveness evaluations are ideally accompanied by process evaluations where the implementation process is monitored and factors that influence implementation are explored in order to provide perspectives to the interpretation of intervention effects, as well as provide important guidance for potential revisions of the intervention and of implementation strategies [16]. Aspects that provide information about the implementation process and that are common to monitor in process evaluations comprise e.g., fidelity (delivery as intended), dose (the quantity of intervention implemented), reach (to what extent the intervention reached the intended target group), and acceptability (reception by participants) [16]. As a process evaluation seeks to add perspectives on how the intervention was carried out and generate an expanded understanding of the function of the intervention, it can benefit from exploring perspectives from several different stakeholders involved or affected by the intervention, and from using both qualitative and quantitative methods combined in a mixed-methods design.

Few prior process evaluations that monitor the implementation of parenting interventions in prison exist. The current literature in the field suggests that important factors for successful implementation of parenting interventions in prison pertain to issues on several levels. On the organisational level, policy, funding and facilities in the correctional institutions influence the successful implementation of interventions [17]. On the participant level, differences in abilities [17] and severity of needs, mistrust towards authorities and participants’ own adverse childhood experiences provide challenges towards intervention implementation [18, 19]. On the parenting intervention level, relevance of the programme material and flexibility in the activities and material are important for successful implementation. The possibility for parent-child contact as part of the programme also influences implementation [20, 21]. Furthermore, aspects related to the programme deliverers, such as their competence, engagement, and work to recruit and retain suitable participants affect the implementation [21].

The process evaluation of FOCS has sought to cover several aspects of the implementation process and perspectives of involved parties. Quantitative monitoring of fidelity of the intervention delivery was carried out both through self-report and observation and was considered acceptable to good. The average dose received was eight out of ten FOCS sessions (unpublished results). In addition, acceptability and factors perceived by the delivering group leaders and responsible correctional inspectors as influencing the implementation of FOCS were investigated in a mixed-methods study using both qualitative and quantitative methods [22]. The study concluded that the high engagement, and the perception that parents had a great need to work on parenting issues, that the deliverers and managers expressed were supportive for the implementation of FOCS. Factors in need of attention to make FOCS sustainable in prison comprised the need for additional resources and support within the overall SPPS [22]. In addition to the perspectives of the deliverers and responsible managers, the perspective of the participating parents is essential to explore in order to understand the possible influence of the parenting intervention. The aim of the present study was to explore parental acceptability of participating in FOCS and how the parents perceived aspects that influenced implementation of the intervention.

## Methods and materials

### Study design

A convergent mixed-methods design (QUAL+ quan) was used in the study to gain a comprehensive perspective from the participating parents with regard to how they perceived participation in FOCS as well as how they perceived factors that influenced the implementation of the FOCS intervention. Quantitative questionnaire data from a larger sample of respondents and qualitative interview data from a smaller sample were collected in parallel in order to capture both a wider and in-depth view of the study aim. Emphasis was given to the qualitative data in this study (QUAL+ quan), and the qualitative findings thus provided a stronger basis for the integrated results and conclusions of the study [23].

### Setting and the FOCS intervention

The FOCS programme aims to support positive parenting for children’s healthy development and targets both mothers and fathers in prison of all security levels in Sweden. All prisons in Sweden are run by the governmental authority The Swedish Prison and Probation Service (SPPS), which has full responsibility for implementing the FOCS intervention. The SPPS acts in accordance with missions stated by the Swedish state and a core focus is to rehabilitate inmates to become part of society as non-criminal individuals. Within the SPPS, women and men are placed in separate prisons whereas inmates serving time for a variety of crimes and time can be placed in the same prison. Inmates with a long sentence are first placed in high security prison, but are, as part of the normal SPPS procedure, moved to lower security prisons during the course of the sentence. The FOCS programme development was a joint effort by the SPPS and the Swedish non-governmental organisation (Barn och ungdom med förälder i fängelse (BUFFF)) and has been described in detail in a published study protocol [15]. FOCS is based on developmental psychology, attachment theory, social cognitive theory, and the Convention on the Rights of the Child (CRC) and is a carried out in groups of 4–10 participants with ten two-hour group meetings delivered by two group leaders. Women and men participate in separate groups whereas inmates convicted for a variety of crimes and time can be included in the same FOCS group. FOCS is delivered in prisons of all security levels. The group leaders follow an intervention manual where each group meeting has a specific theme, e.g., child development and needs across development, the parent’s own childhood, children’s experiences of parental incarceration, and parenting issues [15]. FOCS group leaders complete a five-day-group-leader-training provided by the SPPS. The group leaders subsequently carry out FOCS as part of their main employment in prison, where the majority are employed as prison officers.

### Participants

#### Quantitative sample

The quantitative questionnaire data targeted both general perceptions regarding aspects of importance for implementing parenting interventions in prison, and specific aspects related to FOCS. Therefore, all 85 parents in both intervention and control groups of the FOCS trial were invited to respond to the questionnaire, but the control group only responded to the general items. In total, 46 parents (54%) responded to the questionnaire (22 intervention group, 24 control group), with both men and women in prisons of all security levels. The questionnaire was placed on the last page of a battery of questionnaires and went unnoticed by several of the intervention trial participants which caused the majority of the missing data. Intervention and control group participants had not been matched as group allocation was based on the set operation planning (economic planning for activities one year ahead) at each prison. Thus, the prisons that agreed to participate in the controlled intervention trial and where FOCS was carried out during the period of the trial (in accordance with the operation planning) were included in the intervention group. Prisons that agreed to participate in the trial and that planned for FOCS later were included in the control group. Group leaders recruited participating parents in the prisons that had agreed to participate in the trial.

#### Qualitative sample

As the purpose of the interview data was to provide an in-depth perspective of participation in and implementation of FOCS specifically, only parents from the intervention group were invited to participate in the interviews. A purposeful sample of parents in the intervention group was identified using maximum variation of the characteristics: sex of parent, and prison in the intervention group (all security levels represented). The target sample for the interviews were 15 parents, but due to the covid-19 pandemic possibilities to reach parents in prison were limited and therefore a total of 12 parents were interviewed.

### The consolidated framework for implementation research

The Consolidated Framework for Implementation Research (CFIR) was used to guide both the quantitative and qualitative data collection. The CFIR is a compilation of several implementation frameworks and using the CFIR to guide the data collection enabled us to make use of relevant aspects that influence implementation on multiple levels which have already been identified in the literature. The CFIR [24] identifies 39 specific constructs that have been found to, or hypothesised to, influence outcome in five overarching domains. The domains comprise: 1. Intervention characteristics, which includes constructs that relate to adaptability to local needs, complexity, and design; 2. Outer setting, which includes constructs that relate to patients’ needs and resources, external policy, and incentives; 3. Inner setting, which includes constructs that relate to structural characteristics of the implementing organisation, networks and communication, culture, implementation climate, and readiness for implementation such as leadership engagement; 4. Characteristics of individuals, which includes constructs that relate to knowledge/beliefs about the intervention, self-efficacy, and individual stage of change; and 5. Process, which includes constructs that relate to the planning, execution and evaluation process [24]. This study will focus on the first four overarching domains.

### Data collection

#### Quantitative data

As no existing questionnaire was deemed suitable for the purpose of studying aspects of intervention implementation with parents in a prison context, a new questionnaire was developed for the study, based on CFIR [24], and with a focus on being parsimounious to keep participant burden at a minimum. The authors first derived an initial pool of items. The pool was inspired by two existing questionnaires that had been developed to capture some of the CFIR domains in health care settings [25, 26]. This initial pool of 38 items was subsequently reviewed by experts on implementation research and the SPPS organization, which resulted in a revised pool of 22 items with increased relevance and comprehensibility for the target group. The following step comprised a pilot test of the revised pool with five parents in two prisons to elicit comprehensibility and relevance of the items. Suggestions that arose in the pilot test, accompanied by discussions with experts within the field, resulted in a final scale with seven items for intervention group parents and four items for control group parents. The items targeting intervention group parents covered four of the CFIR domains, ‘intervention characteristics’ with two items, ‘outer setting’ with one item, ‘inner setting’ with two items, and ‘characteristics of individuals/deliverers’ with two items. The items targeting control group parents covered three of the CFIR domains, ‘intervention characteristics’ with one item, ‘outer setting’ with one item, and ‘inner setting’ with two items. All items were responded to on a response-scale from 1—disagree completely, 2—slightly agree, 3 moderately agree, 4 mostly agree to 5—agree completely. The questionnaire was distributed in paper to the parents at the measurement after intervention for both intervention and control groups.

#### Qualitative data

Semi-structured interviews were conducted after the intervention was finished. The interviews were audio-recorded and conducted in Swedish by ÅN (PhD, female, researcher experienced in qualitative methods, and former prison counsellor) using interview guides with open-ended questions based on CFIR [24] (S1 File). Eight of the interviews were conducted via telephone and four were conducted face-to-face in the prison visiting premises and lasted for an average of 51 minutes. The Consolidated criteria for REporting Qualitative research (COREQ (S2 File) has been followed in the reporting of the qualitative data.

### Ethical considerations

Informed consent was provided by all participants in the study. When inviting parents to participate in the study, special attention was paid to the voluntary process of consenting to participate. Parents were given ample time to reflect upon their wish to participate and voluntariness was emphasised. Refusing to participate in the study did not affect the possibility to participate in the FOCS group. The study, including the described consent process, obtained ethical approval from the Swedish Ethical Review Authority 2019–04227.

### Data analysis

#### Quantitative

Analyses of the questionnaire data were undertaken for each specific item as the intervention and control groups had been exposed to items corresponding to their different experience of FOCS (i.e., the intervention group was exposed to items specifically related to FOCS whereas the control group was exposed to items with a general wording). The differences in items also prohibited exploration of internal consistency of the sub-scales corresponding to the CFIR domains of factors influencing implementation. Group means for each item were calculated. When the groups had been exposed to the same items, comparison between groups means were calculated using the non-parametric Mann-Whitney U test. Due to the small group samples, mean comparisons were merely conducted with the intention to gain a broader descriptive view of differences between groups and provide a quantitative view to the qualitative findings in this convergent mixed-method study.

#### Qualitative

Qualitative data were analysed by inductive qualitative content analysis in accordance with the procedure described by Elo & Kyngäs [27]. Data were collected in Swedish and translated into English during the final stages of analysis. Transcriptions were undertaken using an external transcription service. ÅN and PE (PhD, female, researcher, psychologist) conducted the analysis following a stepwise process: first, in order to gain a holistic view of the data ÅN listened to the audio recordings, and both ÅN and PE read the transcripts until a comprehensive view of the data had been established. In the second step, both analysts marked chunks of text that related to the study aim and identified meaning units in the marked text. In the third step, both analysts applied open coding to the meaning units. Then analysts compared their codes and discussed core meanings of different codes to gain a joint understanding of the data and to initiate an understanding of underlying patterns in the data. In the fourth step, ÅN drafted a code book which comprised patterns found in the open codes based on the joint discussion. PE reviewed the code book and applied it to three of the interviews after which a new discussion between the analysts took place. The discussion rendered revision of the code book, after which ÅN applied the code book to all interviews and drafted sub-categories identified from the patterns. In a fifth step, discussion on sub-categories and the merging of sub-categories into generic categories took place. In the sixth and final step, both authors discussed a latent, overarching theme that covered all manifest categories, until consensus was reached. The quotes which illustrate the findings include ellipses, modifications, and explanations within square brackets to enhance comprehensibility. Also, parents who expressed the quotes have been labelled “parent” with a subsequent number to ensure anonymity.

#### Integrated analysis

In a final analytical step, qualitative and quantitative results were merged into a joint display to reflect an in-depth understanding derived from both types of results [23]. This analysis was undertaken by thoroughly reading and comparing the qualitative and quantitative results to gain an understanding of common patterns and essence. This was undertaken by ÅN in an iterative process, where patterns were formed and revised, and reviewed by PE. A joint display was finally created to illustrate joint concepts in both qualitative and quantitative results.

## Findings

Table 1 displays characteristics of the parents who participated in the quantitative and qualitative data. Parents in both qualitative and quantitative data represented prisons on all security levels.

**Table 1:** Characteristics of parents.

Parents	Quantitative data (n = 46)	Qualitative data (n = 12)
Intervention group (% (n))	48% (22)	100 (12)
Women (% (n))	15% (7)	33% (4)
Education: nine years or less (% (n))	41% (19)	33% (4)
Age (mean years (SD))	36.9 (8.9)	31.8 (5.7)
Number of children <18 years (mean)	1.85 (2.0)	1.7 (0.7)
Lived together with the child/ren before conviction (% (n))	74% (34)	83% (10)
Number of convictions (mean (SD))	5.7 (11.2)	8.6 (13.0)
Length of current conviction (mean months (SD))	56.9 (56.5)	63.6 (55.2)

### Quantitative findings

No statistically significant difference between the responders and nonresponders to the quantitative data was found for any participant characteristic.

The intervention group perceived that the prison staff always wanted to do their best in their work to a larger extent compared to the control group (*p* = 0.03, Table 2). No other comparison was statistically significant. Descriptively, the intervention group’s perception was that the prison leadership was more engaged in the FOCS/child perspective (mean: 3.6) than the control group (2.87), although the difference did not reach statistical significance. The intervention group had a positive experience of FOCS where participation was more positive than negative (4.91) and had helped in the parenting work (4.41). In addition, the intervention group scored maximum on group leader engagement (mean: 5.0) and high on their own engagement in the group (mean: 4.86). The control group had a somewhat lower score regarding whether a parenting group could help to improve parenting (mean 3.38), whereas both groups scored equal on their perceived need to work with parenting issues (means: 3.26 and 3.27).

**Table 2:** Results of analysis on quantitative data, descriptive statistics, and group comparisons.

CFIR domain	Mean (SD) per group		Group difference
# item	Intervention (n = 22)	Control (n = 24)	p
Domain: intervention characteristics			
**1**. Participating in FOCS has been more positive than negative (more advantages than disadvantages)^a^	4.91 (0.29)	NR	NA
**2**. FOCS has helped me in my work with my parenting	4.41 (0.91)	NR	NA
**3**. I think that my needs to work with my parenting can be met by participating in a parenting group in prison	NR	3.38 (1.35)	NA
Domain: Outer setting			
**4**. I have a need to work with (get support to improve) my parenting	3.27 (1.16)	3.26 (1.48)	0.97
Domain: Inner setting/the organisation,			
**5**. The prison management supports the implementation of FOCS/work with the child perspective or parenting in prison in a clear and visible way^a^	3.6 ((1.27)	2.87 (1.46)	0.1
**6**. The staff at the prison where I serve time always want to do their best in their work^a^	3.91 (1.23)	3.04 (1.33)	0.03
Domain: Characteristics of individuals/deliverers			
**7**. The group leaders were engaged in the implementation of FOCS^a^	5.0 (0.0)	NR	NA
**8**. I was engaged in the group during the implementation of FOCS^a^	4.86 (0.35)	NR	NA

NR—not reported, i.e., the item was not included in the questionnaire to the group. NA—not applicable (Means: no comparisons between groups can be made as one group was not exposed to the item. Group difference—results of Mann-Whitney U test.

^a^ Response scale 1–5

### Qualitative findings

**Fig 1:**
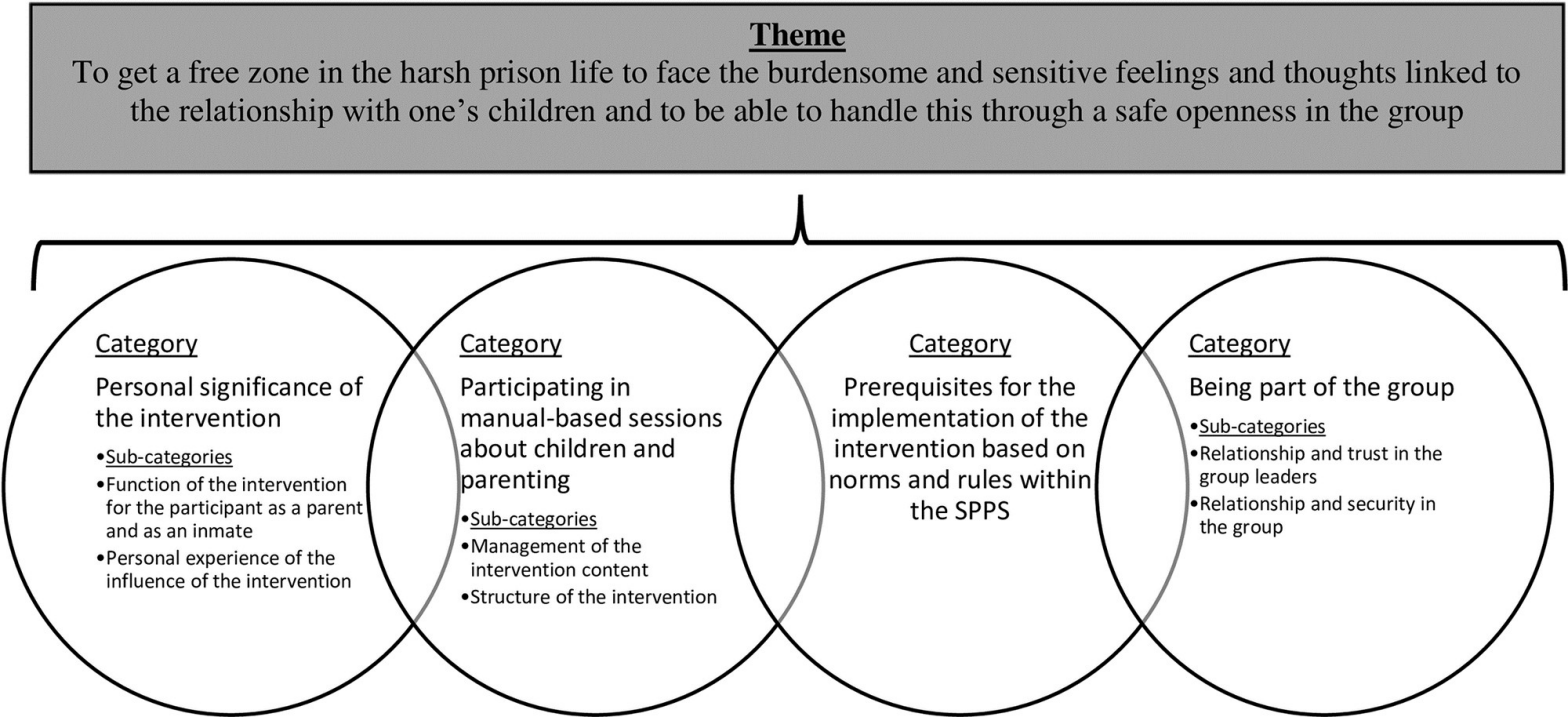
Figure displaying the latent overarching theme above manifest categories and sub-categories.

The analysis of the qualitative data reflected a latent, overarching theme that included all the manifest categories in the data: "To get a free zone in the harsh prison life to face the burdensome and sensitive feelings and thoughts linked to the relationship with one’s children and to be able to handle this through a safe openness in the group" (Fig 1). The theme illustrates how participants struggled to keep reflections regarding the relationship with one’s children and how the own criminality has affected the children, a matter perceived as very burdensome, at bay, where the SPPS’ prevailing norms left little room for children’s perspective, and where harsh attitudes reign among inmates in prison. FOCS then constituted a free zone where it was ok to express difficult experiences, and where participants could feel a sense of fellowship in hearing that others carry the same tough feelings and thoughts. This sense of fellowship, and the, at times, challenging intervention material, generated opportunities to face and process the relationship with one’s children and own parenthood. This was especially rewarding if the group members took the matter seriously, with a warm and open atmosphere. In addition, the group leaders contributed to the atmosphere and opportunities for a reflective process by partly being personal and partly keeping the group to the matter and by being careful when recruiting group members.

#### Personal significance of the intervention

The participants described, in positive terms only, an experience of significance and meaning of taking part in FOCS, partly based on the function that FOCS fulfilled for them in their personal situations both as inmates in prison and as parents, and partly related to the traces that FOCS has left in their lives, through e.g., changed relationships or insights.

*Function of the intervention for the participant as a parent and as an inmate*. The participants described in positive terms how FOCS fulfilled functions for them as both inmates in prisons and as parents, on several levels and beyond the formal purpose stated in the intervention’s manual. As inmates, the participants described that FOCS provided a breathing space in the otherwise harsh prison environment, where the feeling of just being a parent, not an inmate, has prevailed. A respite that served as a reminder about the children, where it was impossible to distract oneself from, or contain, the stressful thoughts and feelings. Instead, this breathing space catalysed thoughts about the importance of changing one’s life to be a better parent.

*“…the problem doesn’t disappear just because you keep distracted […] I could react towards other inmates if they said something*, *[I] was hypersensitive and annoyed*. *And then when we came to [FOCS]*, *sat down and started thinking about these matters*, *then I realised that I had been keeping myself distracted*. *[…T]he [child] matters are really tough*, *but I didn’t touch them kind of [by keeping distracted]*. *So [FOCS] was a break from all the distractions*.*”*

*Parent 1*

As a parent and at the same time prison inmate, FOCS also had the function of creating a sense of fellowship around the stress of being physically away from the children and also to realize how the parent’s own criminality has affected the children. Parents described how they realized that they were not alone in difficulties linked to parenthood, and that they could gain perspectives on and support in their own situations and behaviours, e.g., being honest with their children. Also, getting concrete information about e.g. The CRC and rights and parental obligations served as an important function. Parents also described how FOCS’s primary focus on the children also infused a feeling of the child being present.

*“Sitting there in the parent group talking about your child and others’ children*, *then I felt like [the child] was present”*

*Parent 2*

*Own experience of the influence of the intervention*. The participants described how they, on a personal level, experienced that participating in FOCS had affected them positively in relation to insight into their own childhood, their own parental ability, and understanding of the child. They also described how they felt that FOCS had influenced changes in the relationship with the child and in their own practice of parenthood.

Participants experienced that FOCS generated insights into how their own childhood had affected their life and their own parenting, e.g., repeating patterns of a criminal parent, and what different parenting situations can entail, e.g., to always give children what they want because you want to be kind. At the same time, FOCS generated insight into being valuable as a parent, where participants identified that everything you do for your child is not bad even though you are in prison. Participants also described how they, after FOCS, sought to understand the child’s perspective, experiences, and needs in various situations, e.g., reading the child’s feelings through the tone of voice. The parents identified changes in their own parenting behavior, e.g., by being mentally present with the children on the phone or during visits, listening, asking question, and daring to talk to the child about difficult topics. Parents also described how they let the child’s emotions be present and worked to manage the child’s emotions in a calmer way e.g., by affirming the child’s feelings. This in turn, influenced calmer and closer parent-child relationships, where parents experienced that their presence, calm responses and understanding brought about calmer children, longer parent-child conversations, and increased child trust in the parent.

*“I got the thought that the child may be thinking ‘why do I have to move to [to foster care] and not my sibling*? *Doesn’t [the other parent] want me*?*’ And then I told [the child] ‘I want you to know that I and the social services believed that this was the best for you*. *[The other parent] didn’t want you to move’ And after that [the child] has become calm and confident*. *I think [the child] felt guilty*, *and disappointed in being rejected*. *And I would never have realised those thoughts if I hadn’t joined [FOCS]*.*”*

*Parent 3*

#### Participating in sessions with manual-based material about children and parenting

The participants described in both positive and negative terms how the intervention structure worked and how the content could touch them strongly, and sometimes be demanding to handle.

*Structure of the intervention*. FOCS’s structured sessions, based on themes with a workbook to follow, provided clarity that was perceived as positive. At the same time, the parts of the FOCS’ structure that involved sending home material to share with the child were difficult to pursue for parents if the contact with the co-parent was poor, e.g., as the co-parent may be needed to give the material to the child, or topics that involved the co-parent could be difficult to touch upon. In general, the time allocated to each session was perceived as short at times where some activities had to be excluded as the group needed time to discuss the theme and specific discussion topics in length. To further cater to parent and child needs, participants suggested that the structure of FOCS could integrate family interaction by allowing children and possibly the other parent to participate in some of the sessions, e.g., at the beginning and the end of the programme. In addition, individual meetings with group leaders as a complement to the group meetings, could provide opportunity to talk more openly about sensitive topics, such as severe guilt and shame, own experiences of abuse, or specific problems in the relationship with the child or co-parent.

*“Of course*, *when you work with a theme*, *then feelings and questions arise*. *[And] it is as if the prison gives you crazy notions*, *and you can get bad confidence from the guilt and shame*, *which can hinder change*. *That you don’t dare to say it [in the group]*, *and then individual sessions can be good*.*”*

*Parent 4*

*Management of the intervention content*. Parents perceived the material as stringent and relevant, although it did not always fit the parent’s personal situation, e.g., as the child was of a different age, or the communication with the co-parent was very well functioning without need for improvement. Both the text, but especially the films included in some sessions were seen as very illustrative and apt. Some themes were perceived as heavy and difficult to manage but at the same time very rewarding where films and discussions forced the participants to face stressful insights and feelings about their children’s situation and their own childhood.

*“It was a cartoon*. *The father was taken by the police and the son and daughter blamed themselves… that [session] was the best and the worst*. *That stirred up a lot*. *I recognised myself both as a child and as a father […] That was tough but rewarding at the same time*. *[…] It was tough to feel how I let [my child] down in the same way that I had been let down myself*. *It was in black and white*. *It is exactly the same thing that I expose [my child] to as what I was exposed to myself*.*”*

*Parent 5*

For the themes that were perceived as very heavy to manage, e.g., due to their own experiences of abuse, participants recommended that extra support, e.g., from a psychologist, or group leaders could be helpful or even necessary to prevent drop-out. Furthermore, additional topics such as honour-related oppression, gender equality and more material about children’s emotional development and parenting practices were called for. Also, parents suggested that the material should be adapted to differences in the parental situation experienced by e.g., mothers and fathers.

#### Being part of the group

Being part of the FOCS group signified the importance of a trusting and secure relationship with both group leaders and participants to be able to openly reflect on the sensitive topic of children.

*Relationship and trust in the group leaders*. Gaining confidence in the group leaders in the FOCS group was described as very important for discussions and reflections to be profound and rewarding. The confidence was influenced by various factors, one being the group leaders being parents themselves which enhanced the participants’ ability to relate. Another factor was that group leaders worked in pairs, and if possible, provided perspective from both a mother and a father, and complemented each other in the discussion. Also, having the same group leaders throughout all sessions was essential, as a replacement in a session generated stalled discussions with vigilant participants. Group leaders who thoroughly directed the discussions to focus on the session theme and not on other prison matters, e.g., telephone permits, and who were supportive in discussions with a warm and accepting atmosphere were appreciated and built trust. Group leaders who had a personal attitude and shared their own childhood or parenting experiences were appreciated by participants, whereas group leaders who kept a distance and refrained from showing a personal side, influenced the group to be more restrictive in their exchange of experiences.

*“It’s a confidence*, *that [the group leader] is a little personal and that we are a little personal*. *But they kept a distance*, *because there is a line between a warder and an inmate*, *and I can understand that*. *[But] it would have been helpful for the group [if the group leaders had been more personal]*. *Other than that*, *they were good*, *and good that they were two*, *so that one could continue the discussion if it halted*, *or you didn’t know what to say*. *The most important part was that they were the same two group leaders throughout [FOCS]*. *At some sessions there could be a substitute that no one had seen before and then we just sat and wondered who that person was the entire session*.*”*

*Parent 2*

*Relationship and security in the group*. Being a secure group was emphasized as essential to the participants’ ability to share sensitive topics in discussions with each other. Participants described how the first sessions were tentative in the group interaction but once a feeling of security was established, the group engaged in rewarding and open conversations where all participants were open and respectful of each other, related to different experiences and accepted differences in opinions. At times, e.g., during an emotionally challenging topic such as violence, the group could have difficulties in sticking to the topic. On these occasions, the discussion could become less rewarding if participants turned defensive or aggressive. A safe and open atmosphere was positively influenced by the group composition, e.g., with participants in similar situation, which facilitated interaction and exchange. Barriers to a secure atmosphere were large groups from different wards with participants who did not know each other before, which instead generated a harsh and vigilant attitude. Also, participants with low motivation who were perceived to be present "to have something to do", who spoke in a dishonest manner or simply did not take topics seriously, had a negative influence on the atmosphere. Here, participants highlighted the responsibility and importance of the group leader to be very clear towards participants and to form a secure group. Parents called for group leaders’ to be thorough in the recruitment phase, to form a group where everyone is motivated and focused on the topic, and to be firm in directing participants who do not contribute to the discussion or infuse low motivation during the sessions. Some parents experienced that the group leaders merely picked out participants to fill a group with the result that some participants were unmotivated, which then created a mood of distrust in the group.

*“Three [of the participants] were just bullshit*. *One was quiet the entire time […and] one wasn’t interested and that brings the whole group down*. *The confidence in talking and seeking support decreases*. *[We] set rules*, *and it is sensitive what we talk about*, *it is our kids*, *and in here that can make you lose it*, *if you hear that someone has gone around talking about your child*. *Then the group leaders can’t be soft*, *they should just expel that person immediately*. *That is very important*. *And at the beginning [of FOCS] I didn’t feel secure*, *and considered dropping out actually*, *because there were three participants who were not [in the group] for the right reasons*.*”*

*Parent 6*

#### Prerequisites for the implementation of the intervention based on norms and rules within the SPPS

The opportunities to work on parenting and the child-parent relationship were expressed in the light of underlying norms regarding children and parenting at the SPPS in general, and the actual rules that govern the inmate’s time in the prison specifically. The participants perceived that the general SPPS attitude did not facilitate child-parent contact during the time in prison, illustrated by e.g., long time to get telephone permission, that permission can be denied for reasons that the participants do not feel are linked to the child’s well-being, and that telephone calls are complicated to make once permission is granted. These difficulties led to frustration and hopelessness in relation to maintaining contact with the child.

The participants expressed a general view that the SPPS’ focus on children’s perspectives is weak, and not in accordance with the CRC. Participants expressed that FOCS seemed relatively invisible overall, where FOCS’ existence or the opportunity to participate was relatively invisible in most prisons and that other prisoners had very little knowledge of FOCS existence. Instead, the parents described how they themselves had to seek information, and at times push for FOCS to start. Some participants expressed that they felt lucky that they had a contact person who had knowledge of FOCS, as staff in general did not have much knowledge. At the same time, the fact that FOCS comprises a place with a clear child perspective and that there were cases where other prison staff had knowledge and were positive towards a participant taking time off from the usual prison chores to participate in FOCS, was perceived as very positive.

*“During the prison stay*, *even if there is a focus on establishing contact between the incarcerated parent and the child*, *there is not enough focus on parenting according to me*. *The only time there was a hundred percent focus on parenting*, *and everything that relation to the relationship with the child*, *that was in the [FOCS] group*.*”*

*Parent 7*

### Integrated results

When integrating the qualitative and quantitative results into a joint display (Table 3), three integrated concepts emerged:

**Table 3:** Joint display with integrated understanding of qualitative and quantitative results together.

Integrated understanding	Quantitative results	Int	Cont	Qualitative results	*Theme*
	Domains			Category	
	Items	Mean (SD)	Mean (SD)	Sub-category	
*The importance of a child and parent focused intervention in prison*	Domain: intervention characteristics			Personal significance of the intervention	To get a free zone in the harsh prison life to face the burdensome and sensitive feelings and thoughts linked to the relationship with one’s children and to be able to handle this through a safe openness in the group
	**1**. Participating in FOCS has been more positive than negative (more advantages than disadvantages)	4.91 (0.29)	NR	• Function of the intervention for the participant as a parent and as an inmate	
	**2**. FOCS has helped me in my work with my parenting	4.41 (0.91)	NR	• Personal experience of the influence of the intervention	
	**3**. I think that my needs to work with my parenting can be met by participating in a parenting group in prison	NR	3.38 (1.35)	Participating in a manual-based sessions about children and parenting	
	Domain: Outer setting			• Management of the intervention content	
	**4**. I have a need to work with (get support to improve) my parenting	3.27 (1.16)	3.26 (1.48)	• Structure of the intervention	
*Partly unsupportive organisational norms with irregular staff engagement*	Domain: Inner setting/the organisation,				
	**5**. The prison management supports the implementation of FOCS/work with the child perspective or parenting in prison in a clear and visible way	3.6 (1.27)	2.87 (1.46)	Prerequisites for the implementation of the intervention based on norms and rules within the SPPS	
	**6**. The staff at the prison where I serve time always want to do their best in their work	3.91 (1.23)*	3.04 (1.33)*		
*The importance of engagement and motivation from all participants and group leaders in the group*	Domain: Characteristics of individuals/deliverers			Being part of the group	
	**7**. The group leaders were engaged in the implementation of FOCS	5.0 (0.0)	NR	• Relationship and trust in the group leaders	
	**8**. I was engaged in the group during the implementation of FOCS	4.86 (0.35)	NR	• Relationship and security in the group	

NR—not reported, i.e., the item was not included in the questionnaire to the group,

* significant group difference p< 0.05—results of Mann-Whitney U test

#### The importance of a child and parent focused intervention in prison

This joint concept comprises the qualitative findings in the categories *personal significance of the intervention* and *participating in manual-based sessions about children and parenting*, and the quantitative domains *intervention characteristics* and *outer setting*. The qualitative findings reflect that FOCS had several positives functions beyond the intended ones, where parents also described how their lives had been positively influenced by FOCS, and that, although some of the sessions were very tough to attend with sensitive issues to face, participating in FOCS had been rewarding. The quantitative results show that all intervention group parents scored high with regard to if FOCS is positive and helpful. The scores of both groups reflect a perception that the need to work with their parenting is moderate (mean: 3.26–3.27).

#### Partly unsupportive organisational norms with irregular staff engagement

This joint concept comprises the qualitative findings in the category *prerequisites for the implementation of the intervention based on norms and rules within the SPPS* and the quantitative domain *inner setting*. The qualitative results reflect parents’ views that child and parenting issues are treated at the margin by the SPPS, which frustrates the parents, and seems to make FOCS somewhat invisible in prison. This is mirrored by the quantitative scores where both groups had a partial to disagreeing perception (mean: 2.87–3.6) about the prison management being supportive towards FOCS or the child perspective. At the same time, parents voice that engaged prison staff are perceived as supportive, and this irregularity is also shown in the quantitative results where the intervention group scored significantly higher on the perception that the prison staff wanted to do their best in their work.

#### The importance of engagement and motivation from all participants and group leaders in the group

This joint concept comprises the qualitative findings in the category *being part of the group* and the quantitative domain *characteristics of individuals*. The qualitative results reflect the sincere importance of creating trustful relationships in a group that includes both parents and group leaders who take the matter seriously and take responsibility for their roles in the group. The parents contribute with their experiences, thoughts and feelings in the discussions and group leaders keep the discussions on track, being thorough in recruiting and retaining parents who are interested and engaged. The quantitative results also depict high intervention group score on group leader and own engagement.

## Discussion

This study explored perceptions of participating in, and aspects that influenced implementation of the FOCS parenting intervention from the perspective of participating parents. The integrated analysis of the qualitative and quantitative findings identified that the availability of FOCS in prison was of vital importance to parents, but that a rewarding participation depended on all participants’ and group leaders’ high motivation and interest in the group. It also showed that the organisational norms of the SPPS were perceived as unfit for implementing FOCS, although when individual prison staff were engaged in FOCS, this was considered helpful. The findings from this study can contribute to further improvement of implementation strategies for interventions in prison, both specifically focusing on parenting and child issues, but also interventions with a personal or behavioural change focus beyond the one of FOCS, carried out in the prison setting.

### The importance of a child and parent focused intervention in prison

The findings highlight how the availability of an intervention that focuses on child and parenting issues is of great importance to parents in prison which is signalled by parents’ perceptions of how FOCS has influenced them, the positive functions of FOCS, and the ratings of support and advantages of FOCS. International research has shown that parenting interventions can have effects on parenting [12–14], and child outcomes [13]. The effectiveness evaluation of FOCS found significant effects on parent-child relationship and interest in further engaging in treatments that focus on criminality (unpublished results). In addition, both group leaders and correctional inspectors involved in FOCS emphasise that working with parenting and child issues for incarcerated parents is important and fills a great need [22]. Thus, FOCS seems to fill a gap of needs and has a positive influence on the lives of incarcerated parents in prison in Sweden. However, parents also suggest extension of FOCS to further cater to the needs of managing the profound and sensitive issues that parent-child relationships and parenting entail, e.g., by adding interaction with the child and family, include individual sessions to manage personal issues related to children and parenting in-depth, and additional themes such as how to engage in positive parenting practices. Suggestions to extend the work on parent-child relationships and parenting are in line with prior research which suggest that interventions to prevent the intergenerational effect of criminality should be targeting family factors, where positive parenting is emphasised [9, 28]. Thus, extensions of FOCS may benefit not only the parents in their immediate strive towards changing their parenting and improving the relationship with their child, but also the children’s healthy development across childhood. It is well documented in the literature that children with incarcerated parents run a higher risk of a number of negative health outcomes, marginalisation and own delinquency [4–9]. An increased focus on, and extension of FOCS may therefore contribute to improved health, less marginalisation and prevention of crime and suffering over time in this child group. This chain of intervention effects on outcomes has been hypothesised for FOCS specifically and was described in the published study protocol [15]. Furthermore, at present FOCS is not viewed as part of the treatment programmes within the SPPS which are designed to have an impact on a criminal lifestyle. Instead FOCS is viewed as an extra activity that inmates are offered just because they find it interesting. This is also evident by the organisational affiliation of FOCS within the authority, where FOCS is not placed in the treatment programme structure with the result that FOCS is not prioritised and does not generate funding for the prisons. Thus, from the perspective of the SPPS, FOCS is not expected to have an impact on the lifestyle changes towards a non-criminal lifestyle. However, FOCS may fit well into a structure of rehabilitation programmes by focusing on building healthy relationships. For individuals with criminal behaviours, the recreation of social bonds that focus on healthy relationships, where prosocial behaviour is favoured, can provide a route towards reduced recidivism. This chain of behaviour change towards a non-criminal lifestyle has been proposed by international studies [29–32] and by the effectiveness evaluation (unpublished results) and serves as a firm foundation for the contribution of a parenting/child focused intervention such as an extended version of FOCS in the rehabilitations structure to reduce recidivism.

### Partly unsupportive organisational norms with irregular staff engagement

The study findings highlight the perception of child and parenting issues as being at the margin of the SPPS where FOCS ends up rather invisible, and the rather low score on prison management being supportive towards FOCS or the child perspective, but that occasional engaged prison staff apart from the group leaders are perceived as helpful. These findings echo the perceptions of group leaders and correctional inspectors who voiced the lack of resources allocated to FOCS by the SPPS on a several levels, including the structural level which results in lack of economical and staff resources [22]. The resource situation made group leaders and correctional inspectors describe it as a lonely struggle to implement FOCS, where it was up to them as persons, and their personal interest and engagement in the importance of working with parenting in prison, to made FOCS come about in their different prisons [22]. Previous studies have found that prison policies where parenting issues are marginalised, and high demands on prison staff can have negative influences on intervention implementation in prison [17, 33, 34]. The general implementation literature highlights that available resources are essential for successful intervention implementation, where resources available to carry out the intervention have been linked to beneficial outcomes [35] and stable funding constitutes an important factor for ensuring intervention sustainability [17]. Organisational attitudes such as giving the intervention an efficient place within the organisational structure, organisational willingness to take on an intervention, and leadership engagement comprise important factors for successful implementation of an intervention [24, 35, 36]. The seemingly unfit organisational norms and the lack of resources allocated to FOCS, may contribute to potentially makes FOCS less accessible to inmates, as FOCS is described as rather invisible in the prisons. Thus, increasing focus and resources allocated to FOCS may be beneficial to increase parental participation and positive effects in parent-child relationships and outcomes related to changing criminality in accordance with the effectiveness evaluation (unpublished results). The work of the SPPS is guided by the mission pronounced by the state of Sweden and at its core, the SPPS, works to reduce recidivism. However, in the work to reduce recidivism, rehabilitation is a key strategy which is materialised in the form of studies, vocational training, and CBT-based rehabilitation programmes in prison. The building of healthy and strong relationships e.g., with close family members, has been acknowledged to some extent in the rehabilitation strategies within the SPPS but are often overlooked in practice. Including such focus in the mission from the Swedish state could increase the allocation of resources and emphasise the need for a shift in norms within the SPPS around child and parenting issues. This could in turn facilitate the building of a firm structure and prioritisation of parenting and child issues, making FOCS more accessible to parent inmates.

### The importance of engagement and motivation from all participants and group leaders in the group

The findings show that it is essential to create trustful relationships within the group, with both parents and group leaders and where the group leaders are thorough in fulfilling their role of keeping the group discussions and participants on track. These findings can be viewed in the light of previous findings where the group leaders also identified the importance of creating a safe space in the group, but where they found it very challenging to hold a group with such heterogeneous needs together and keep a focus on the theme at the same time. Group leaders described how they managed participants with often heavily marginalised backgrounds and current life situations and also with a widespread vigilance towards authorities. At the same time, they were to steer the group through sensitive topics that may stir up intense emotions and related behaviours such as aggressiveness [22]. The importance of having the skill and competence to create such safe space and direct the group in order to facilitate implementation of parenting interventions in prison has been found in the intervention literature [19, 21]. The group leaders and responsible correctional inspectors also voiced the need to further enhance competence and skills among group leaders [22], which could be considered an important financial post if additional resources are to be allocated to FOCS or in the development and revision of the implementation structures of similar prison interventions. This study also highlights the importance of highly skilled and experienced group leaders. The parents voice firmly that if group leaders do not take careful responsibility for all parts of planning, recruiting and interaction with the group, the group could turn into something uncomfortable or even severely intimidating (e.g., spreading rumours about the parent and child among other inmates). This parental experience should be taken into regard in the further work to implement FOCS and related interventions in the prison context.

Furthermore, the issue of the harsh atmosphere among inmates at large in prison is raised on several occasions in the qualitative findings. Overcoming this harsh, vigilant atmosphere emerged as an uttermost important factor for success in getting a rewarding experience from FOCS participation. Previous studies have found that participation in educational programmes in prisons can mitigate maladaptive behaviours among inmates and contribute to a safer prison environment [37], which further points towards a beneficial circle of implementation FOCS in prison. Importantly though, as mentioned above parents in this study describe that the group may also have a negative effect on communication and relations between inmates if the group is not managed properly by the group leaders. Hence, a poor group atmosphere may contribute to more conflicts in the prison environment which points further to the importance of highly skilled group leaders. Thus, the importance of managing the otherwise harsh between-inmate atmosphere should be further acknowledged an emphasised in the development of implementation strategies to further successful implementation of FOCS or similar interventions in prison. In addition, the development of items to be included in a quantitative scale to measure this atmosphere should be considered, as it was not included in this study.

### Strengths and limitations

A strength of the study comprises the rigor with which the qualitative data was processed. In order to ensure trustworthiness, the audit-trail of the process has been thoroughly described, and the analysis included co-coding and peer-review with two researchers. In addition, the results have been described with information rich quotes to illustrate the findings [38, 39]. The application of a mixed-methods design which includes both qualitative and quantitative data comprises a strength of the study as it provides a holistic view of participation in, and implementation of, a parenting intervention in the prison setting. A number of limitations of the study are connected to the questionnaire data which is also acknowledged by the emphasis on the qualitative data described in the methods section of this study as (QUAL + quan). The limitations pertain to: first, the study comprises the limited possibility to compare questionnaire responses between the intervention and control groups. The limited sample of respondents who responded to the questionnaire comprises yet another limitation. The sample does not allow for any thorough psychometric evaluation of the questionnaire which was desired as the questionnaire was newly developed. In addition, the questionnaire only comprises a small number of items, where some CFIR domains are only represented with one item and thus may not capture the complexity of the CFIR domain. Also, the intervention and control groups did not respond to the same items for all CFIR domains included in the questionnaire which limits the possibility for statistical comparisons between the group.

## Conclusion

This study shows that FOCS seems to fill an intensely needed function of space where the inmates can let loose their inner world related to the profound and severely sensitive relationship with the children and role of being a parent. The demanding task of creating a trustful relationship in the group, which depends on several factors related to thoroughness in recruitment, and group leader competence and skill in keeping the group on track, building rapport, and infusing an atmosphere of trust seem to require additional attention in order to be a self-evident part of implementing FOCS. Findings of this study can guide further implementation and development of child and parent focused or other similar interventions in prison.

## Supporting information

S1 File Interview guide. (PDF)

S2 File COREQ checklist. (PDF)

## Abbreviations

CFIR: The Consolidated Framework for Implementation Research
CRC: Convention on the Rights of the Child
FOCS: For Our Children’s Sake
SPPS: Prison and Probation Services
