# Supplementary material for: Participating in a parenting intervention in prison, perceptions from incarcerated fathers and mothers—A convergent mixed-methods study
Source: PLoS One. 2023 Mar 1;18(3):e0282326. doi: 10.1371/journal.pone.0282326 (PMC9977051; doi:10.1371/journal.pone.0282326)
Supplement: S1 File — (PDF) [file pone.0282326.s001.pdf]

Supporting information 1. Interview guide

Interview guide for parents

- How did you experience that "For of our children’s sake" worked in general?
- What made you choose to participate in "For of our children’s sake"?
- What parts of "For of our children’s sake" worked well/less well?
- How did you perceive the work with the different themes?
    - What was good/less good?
    - How were the themes adapted to your specific parenting situation?
    - What type of adaptation would be needed to increase the relevance further?
- What would you have needed to facilitate the work with "For of our children’s sake"?
- How did you use the work with "For of our children’s sake" in the contact with your child?
- How did you perceive working with "For of our children’s sake" together with the others in the group?
- How do you perceive the engagement from the group leaders?
- How do you perceive that surrounding factors at the prison influenced the work with "For our children’s sake”?
- What would you need to continue the work from "For our children’s sake”?
- What would you like to do differently if you could do "For of our children’s sake” again?
